# Molybdenum Carbide Anchored on N,S Co-Doped Carbon Composite Derived from Lignosulfonate as a High Performance Electrocatalyst for Hydrogen Evolution Reaction

**DOI:** 10.3390/nano12173047

**Published:** 2022-09-02

**Authors:** Na Yeong Oh, So Young Park, Ji Young Hwang, Hyung Mo Jeong, Yong Sik Kim, Duck Hyun Youn

**Affiliations:** 1 Department of Chemical Engineering, Department of Integrative Engineering for Hydrogen Safety, Kangwon National University, Chuncheon 24341, Korea; 2 School of Mechanical Engineering, Department of Smart Fab. Technology, Sungkyunkwan University, Suwon 16419, Korea; 3 Department of Paper Science & Engineering, Kangwon National University, Chuncheon 24341, Korea

**Keywords:** electrocatalyst, hydrogen evolution reaction, lignosulfonate, molybdenum carbide, N,S co-doped carbon

## Abstract

A composite of Mo_2_C nanoparticles dispersed onto a nitrogen and sulfur co-doped carbon scaffold (Mo_2_C/N,S-C) was prepared by a simple and environmentally friendly method of one-pot annealing of MoCl_5_, urea, and lignosulfonate under a N_2_ atmosphere at 700 °C. Lignosulfonate, a by-product of the sulfite pulping process, was employed as a feedstock to fabricate the S-doped carbon scaffold and carbide simultaneously, and urea acted as a nitrogen source for N-doping to carbon. The as-prepared Mo_2_C/N,S-C catalyst showed high performance for the hydrogen evolution reaction (HER), with a small overpotential of 105 mV at 10 mAcm^−2^, and good stability for 3000 cycles. The improved HER performance of the Mo_2_C/N,S-C originated from the interplay between the highly active Mo_2_C nanoparticles and the N,S co-doped carbon scaffold with its high electrical conductivity and large surface area. Furthermore, N,S co-doping to carbon improved the hydrophilicity of the catalyst surface, thus further enhancing the HER activity.

## 1. Introduction

Increasing energy consumption and environmental pollution have prompted the development of clean and renewable energy sources for the alleviation of human dependence upon exhaustible fossil fuels [1,2]. Hydrogen is recognized as an alternative energy source as it is abundant, clean, and energy-dense. Water electrolysis powered by renewable electricity is one of the most environmentally benign and sustainable hydrogen production technologies [3,4,5]. A hydrogen evolution reaction (HER) is the cathodic reaction of water electrolysis, wherein Pt is the most efficient electrocatalyst due to the negligible overpotential provided by the appropriate hydrogen binding energy of its surface [6]. However, the high cost and finite reserves of Pt limit large-scale application of water electrolysis. Therefore, the development of noble-metal-free electrocatalysts is of great importance [7,8].

Various molybdenum-based catalysts, including carbides (Mo_2_C) [9,10], sulfides (MoS_2_) [11,12], nitrides (Mo_x_N, Ni_2_Mo_3_N) [13,14], and phosphides (MoP) [15], have proven their good HER activity as possible replacements for Pt. In particular, molybdenum carbides are attracting tremendous attention due to their high activity and stability for HER from their Pt-like electronic structure, high electrical conductivity, and high chemical stability [16,17]. Since Vrubel and Hu revealed that commercial Mo_2_C possesses HER activity in both acidic and alkaline media [18], extensive research has been carried out to enhance the HER performance of Mo_2_C by fabricating nanostructured Mo_2_C catalysts and their composites with carbonaceous materials [19,20,21,22,23,24,25,26].

Next to cellulose, lignin is the second most abundant biopolymer and is generated as a by-product of the pulping process [27,28]. The lignin becomes sulfated during the sulfite pulping process, and the lignosulfonate is produced as a cross-linked polyphenolic polymer that contains sulfonic acid groups [29]. In contrast to hydrophobic lignin, lignosulfonate is an amphiphilic biopolymer and, as such, is soluble in water [30]. Small portions of produced lignosulfonate have been used as surfactants and adsorbents; however, most of it is unutilized and combusted for disposal producing carbon dioxide [31]. Thus, the effective utilization of lignosulfonate is required and inspired by its high carbon content and the presence of sulfur, we regard it as a suitable feedstock for the simultaneous fabrication of heteroatom-doped carbon and carbide.

Herein, sulfur and nitrogen co-doped carbon scaffolds decorated with molybdenum carbide nanoparticles (Mo_2_C/N,S-C) were fabricated via the one-step pyrolysis of MoCl_5_, urea, and lignosulfonate at 700 °C under N_2_ flow for HER. During the synthesis, lignosulfonate plays critical roles as a source of sulfur for S-doping to carbon, and a source of carbon for the formation of amorphous carbon scaffold and carbide. At the same time, urea was employed as a nitrogen source for N-doping to carbon. Indeed, molybdenum carbide nanoparticles on S-doped carbon (Mo_2_C/S-C) was fabricated without urea. The resultant Mo_2_C/N,S-C catalysts exhibited excellent HER activity in alkaline solution with a low overpotential value of 105 mV at 10 mA cm^−2^, which is better than Mo_2_C/S-C and commercial Mo_2_C (c-Mo_2_C). Furthermore, the Mo_2_C/N,S-C showed good stability for 3000 cycles. The enhanced HER performance of Mo_2_C/N,S-C is due to the interplay between highly active Mo_2_C nanoparticles and N,S-C scaffold providing high surface area and electrical conductivity. In addition, the enhanced HER performance of Mo_2_C/N,S-C is assisted by the improved hydrophilicity of the N,S-C scaffold relative to S-C scaffold. The high electrocatalytic performance and the simple and environmentally friendly synthetic method suggest that the Mo_2_C/N,S-C could be a promising catalyst for HER.

## 2. Materials and Methods

### 2.1. Mo_2_C/N,S-C Synthesis

An amount of 160 mg lignosulfonate (Aldrich, Pittsburgh, PA, USA) was dissolved in 15 mL ethanol under magnetic stirring. One gram of MoCl_5_ (Alfa aesar, Haverhill, MA, USA) was dispersed in 2.53 mL ethanol and added to the lignosulfonate solution under vigorous stirring for 30 min. Then, 109.9 mg urea was added as a nitrogen source, with a molar ratio (R) of 0.5 with respect to Mo and stirred for 1 h. After drying the solution in an oven at 100 °C for 90 min, the resultant mixture was annealed at 700 °C (3 °C min^−1^ ramp) for 3 h under a N_2_ atmosphere. As a control experiment, molybdenum carbide on a S-doped carbon (Mo_2_C/S-C) catalyst was prepared by an identical method, except that 250 mg lignosulfonate was employed without the addition of urea. The weight contents of Mo_2_C were measured as 35~40 wt.% for both the Mo_2_C/N,S-C and Mo_2_C/S-C samples.

### 2.2. Catalyst Characterization

Field-emission transmission electron microscopy (FE-TEM, JEOL, Akishima, Japan, JEM-2100F) and field-emission scanning electron microscopy (FE-SEM, JEOL, JSM-7900F) with energy dispersive X-ray spectrometer (EDS) were used to analyze morphologies and elemental compositions of the prepared samples. X-ray diffraction (XRD, Rigaku, Tokyo, Japan, MiniFlex 600) was conducted with Cu Kα (1.54 Å) radiation. X-ray photoelectron spectroscopy (XPS, Thermo-Scientific, Waltham, MA, USA, K-alpha) was used to investigate the chemical states of samples. The obtained binding energy values were calibrated by referencing the C 1s peak at 284.4 eV. The specific surface area and corresponding pore size distribution were investigated by measuring the N_2_ adsorption–desorption isotherms at 77 K (Micromeritics, Norcross, GA, USA, ASAP 2020 PLUS). The contact angle measurements (Kruss, Kruss, Germany, Germany, DSA25) were conducted by loading the prepared catalysts (1 mg cm^−2^) onto 1 × 1 cm carbon paper (1 wt.% wet-proofing, Toray, Tokyo, Japan, TGP-H-060).

### 2.3. Electrochemical Tests

All of the electrochemical measurements were conducted on a three-electrode electrochemical workstation (PAR, VersaSTAT 4) equipped with a rotating disk electrode (RDE, PINE Research) in a 1 M KOH aqueous solution. To prepare the working electrode, 20 mg of prepared catalyst was dispersed in 2 mL water and then 20 μL catalyst ink was loaded onto a glassy carbon electrode (0.19635 cm^2^). The reference and counter electrodes were Ag/AgCl (4 M KCl) electrode and Pt wire, respectively. All recorded potential values were converted to the reversible hydrogen electrode (RHE) with iR-compensation. Linear sweep voltammetry (LSV) curves were conducted at scan rate of 5 mV s^−1^ with 900 rpm. Electrochemical impendence spectra (EIS) were performed in the frequency range from 100 kHz to 0.1 Hz at 105 mV (vs. RHE) overpotential with 6 mV modulation amplitude. Stability tests of prepared electrocatalysts were obtained by repeating 3000 cycles with potential range of 0.2 V to −0.2 V (vs. RHE). Electrochemical double layer capacitances (EDLC) were conducted by cyclic voltammetry (CV) from 0.1 to 0.3 V (vs RHE) at various scan rates of 20, 60, 100, 140 and 180 mV s^−1^

## 3. Results and Discussion

**Scheme 1:**
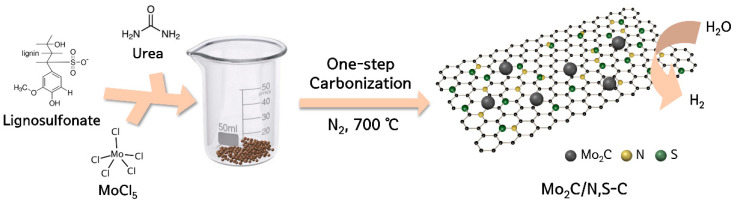
Schematic illustration for the preparation of Mo_2_C/N,S-C.

The synthesis of the Mo_2_C/N,S-C catalyst is summarized in Scheme 1. Molybdenum chloride and urea was dissolved in ethanol solution containing the lignosulfonate and the subsequent annealing under N_2_ at 700 °C yielded Mo_2_C/N,S-C. During the synthesis, urea acted as a nitrogen source and lignosulfonate acted as carbon and sulfur sources. Notably, Mo_2_C/S-C catalyst was generated without urea by an identical synthetic method, thereby demonstrating that that carbon sources from lignosulfonate played multiple roles in generating carbide and carbon scaffold at the same time. In addition, sulfur and nitrogen sources from lignosulfonate and urea served to S and N doping to carbon scaffold, respectively. The proposed synthetic method has the following advantages: (i) it is a simple method in which the formation of Mo_2_C and the generation of amorphous carbon scaffold and N,S co-doping to the carbon scaffold are synchronously accomplished via mixing and annealing of the precursors in one pot, (ii) no toxic gases or chemicals were required for Mo_2_C synthesis and N,S co-doping, (iii) the method is environmentally friendly due to the use of lignosulfonate, an industrial waste material, as a precursor, and (ⅳ) our synthetic method produced Mo_2_C nanoparticles with a size of ca. A total of 7 nm dispersed in N,S co-doped carbon scaffold (Mo_2_C/N,S-C), which recorded one of the best performances for HER among the biomass-derived Mo_2_C-based catalysts.

**Figure 1:**
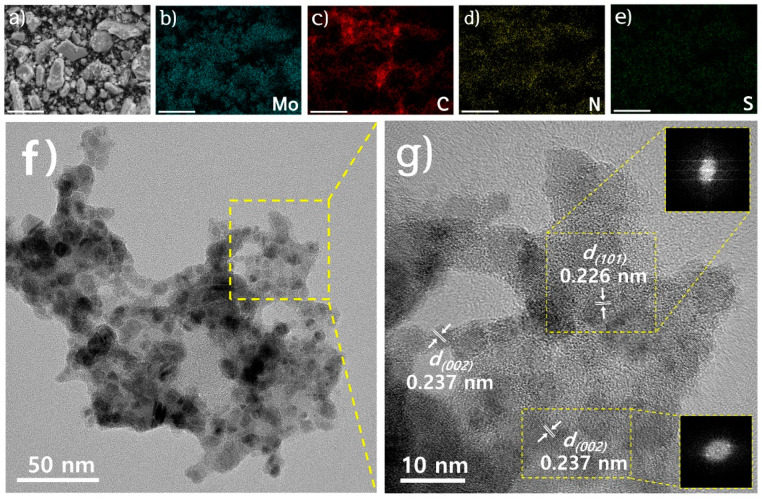
(**a**) SEM image of Mo_2_C/N,S-C and corresponding SEM-EDS elemental mapping for (**b**) Mo, (**c**) C, (**d**) N, and (**e**) S (scale bar = 10 um). (**f**) TEM and (**g**) HR-TEM images of Mo_2_C/N,S-C. Insets in (g) denote FFT images.

The SEM image of the Mo_2_C/N,S-C in Figure 1a reveals the presence of amorphous carbon clusters of ~4 um in size, while the corresponding EDS elemental mapping images in Figure 1b–e reveal the almost identical distributions of molybdenum, carbon, nitrogen, and sulfur, respectively, thereby indicating that the Mo_2_C nanoparticles are uniformly distributed on the N,S co-doped carbon scaffold. In the TEM image (Figure 1f), the Mo_2_C nanoparticles are dispersed on amorphous carbon composite with an average particle size of 7 nm. The HR-TEM and fast Fourier transform (FFT) images are shown in Figure 1g. The lattice spacings of 0.226 and 0.237 nm are assigned to Mo_2_C (101) and (002) crystalline planes, respectively (Figure 1g). In comparison, TEM images of Mo_2_C/S-C were also presented in Figure S1. The Mo_2_C nanoparticles are distributed on S-doped carbon scaffolds with a diameter of 16 nm. The lattice distance of 0.237 nm corresponds to the reflection of the (002) plane (Figure S1).

**Figure 2:**
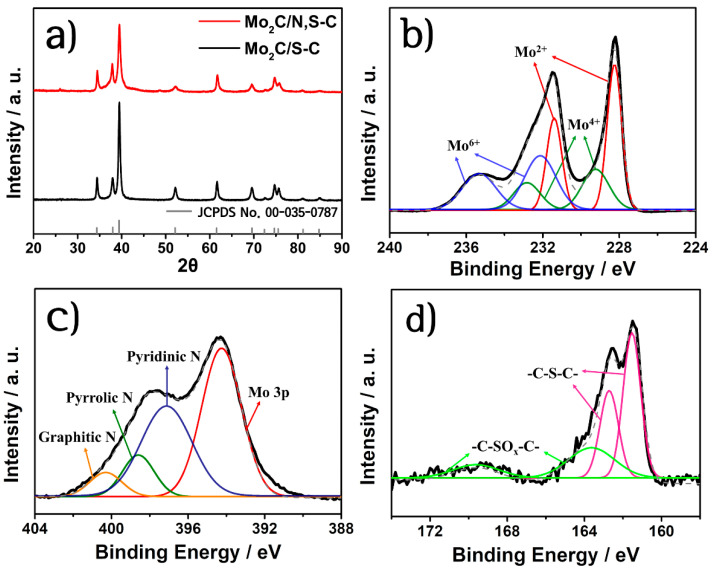
(**a**) XRD patterns of Mo_2_C/N,S-C and Mo_2_C/S-C. (**b**–**d**) High-resolution XPS spectra of Mo_2_C/N,S-C for Mo 3d, N 1s and S 2p. Dotted lines denote raw data and other lines represent deconvoluted data.

Figure 2a shows the XRD patterns for the Mo_2_C/N,S-C and Mo_2_C/S-C catalysts, which are consistent with their hexagonal β-Mo_2_C reference XRD patterns (JCPDS 00-035-0787). The peaks at 34.4°, 38.0°, 39.4°, 52.1°, 61.5°, 69.6°, 74.6°, 75.5°, 81.2° and 84.8° correspond to the (100), (002), (101), (102), (110), (103), (112), (201), (004), and (202) lattice planes, respectively. No impurity peaks were detected such as metallic molybdenum or molybdenum oxides for both catalysts.

The XPS survey spectrum for Mo_2_C/N,S-C (Figure S2) confirmed the existence of molybdenum, nitrogen, carbon, and sulfur elements on the catalyst surface. The amounts of N and S in Mo_2_C/N,S-C due to co-doping to carbon scaffold were determined to be 3.18 and 0.72 at.%, respectively, while Mo_2_C/S-C (Figure S3a) contained 0.49 and 0.9 at.% of N and S. The elemental analysis results in Table S1 suggest that the N in the Mo_2_C/S-C is derived from the lignosulfonate itself; however, the doping amount of N in Mo_2_C/S-C is nearly 6.5 times lower than that in Mo_2_C/N,S-C. The amounts of each element for Mo_2_C/N,S-C and Mo_2_C/S-C are summarized in Table S2. 

In the high-resolution Mo 3d spectrum (Figure 2b), the peak deconvolution affirmed the existence of Mo_2_C (Mo^2+^ at 228.25/231.4 eV), MoO_2_ (Mo^4+^ at 229.2/232.8 eV), and MoO_3_ (Mo^6+^ at 232.1/235.3 eV) [32,33,34]. The presence of oxides on the surface of the carbide material is unavoidable due to air exposure [9]. The N 1s spectrum in Figure 2c shows four peaks at 394.3, 397.1, 398.6, and 400.3 eV, corresponding to Mo 3p, pyridinic N, pyrrolic N, and graphitic N, respectively. There are two peaks at S 2p spectrum for Mo_2_C/N,S-C shown in Figure 2d. The peaks at 161.5/162.68 eV were related sulfur doping to the carbon supports, and the peaks for oxidized S was determined at 163.6/169.6 eV [35,36]. The Mo 3d and S 2p spectra of Mo_2_C/S-C were similar to those of Mo_2_C/N,S-C (Figure S3b,d), thereby suggesting similar chemical states except for the much lower content of N element (Figure S3c).

The mesoporous texture of the Mo_2_C/N,S-C was revealed by the nitrogen adsorption-desorption results shown in Figure S4a, which exhibit a type IV isotherm. The Brunauer–Emmett–Teller (BET) surface area of Mo_2_C/N,S-C is 34.4 m^2^ g^−1^ and the presence of mesopores was also verified by pore size distribution (PSD) using the Barrett–Joyner–Halenda (BJH) method (Figure S4b).

**Figure 3:**
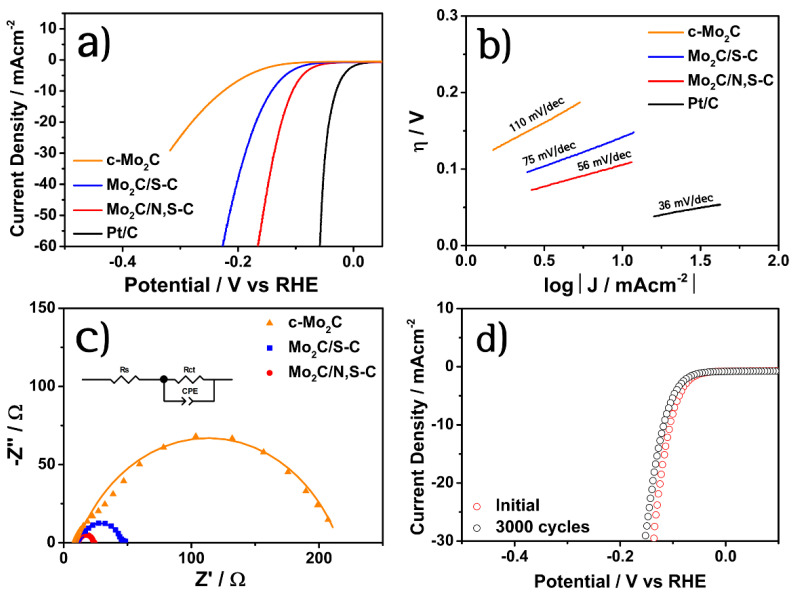
(**a**) Polarization curves of all prepared samples in 1 M KOH solution, (**b**) Tafel plots, (**c**) Nyquist plots with an equivalent circuit, and (**d**) stability tests of Mo_2_C/N,S-C.

**Figure 4:**
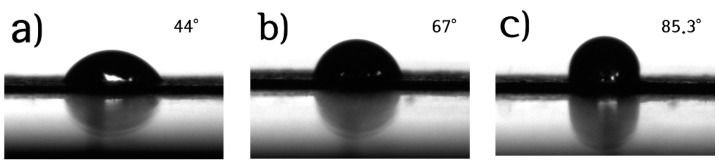
Contact angle measurement for (**a**) Mo_2_C/N,S-C, (**b**) Mo_2_C/S-C, and (**c**) c-Mo_2_C.

Figure 3a shows polarization curves for the prepared catalysts including Pt/C (20 wt.%, E-TEK) and commercial Mo_2_C (c-Mo_2_C) in 1M KOH solution. The Pt/C exhibited the best HER activity with small overpotential of 30 mV to drive 10 mA cm^−2^ (η_10_ value) [14]. By contrast, the c-Mo_2_C with the η_10_ value of 225 mV is not suitable as a HER electrocatalyst which might be due to its largely aggregated particles and low electrical conductivity [18]. In contrast, the Mo_2_C/S-C exhibited an improved HER activity with η_10_ value of 141 mV than c-Mo_2_C, and this was significantly enhanced when the Mo_2_C phases are combined with the N,S co-doped carbon scaffold, achieving a η_10_ value of 105 mV for the Mo_2_C/N,S-C. This improved performance is comparable to that of the previously reported biomass-derived Mo_2_C-based catalysts (Table S3). The coupling of Mo_2_C with N,S-C scaffold not only prevented agglomeration of Mo_2_C particles, but also provided high surface area and electrical conductivity [35]. In addition, the co-doping of N and S increases the charge and spin densities of carbon atoms compared to solely the doped carbon scaffolds, which results in a larger number of active carbon atoms [37]. Thereby, the N,S-C scaffold can further enhance the HER performance of Mo_2_C by modulating the catalytic activity of carbon atoms adjacent to heteroatoms [38]. In addition, previous studies have demonstrated that multi-heteroatom doping increases the surface wettability of the electrocatalysts, thereby promoting their HER activity [39,40,41,42]. Hence, the increased activity of the Mo_2_C/N,S-C relative to the Mo_2_C/S-C is further explained in terms of the surface wettability of the catalysts in Figure 4. The water contact angles of the Mo_2_C/N,S-C, Mo_2_C/S-C, and c-Mo_2_C are found to be 44°, 67°, and 85.3°, respectively, thereby demonstrating that hydrophilicity was greatly enhanced by the introduction of N,S co-doped carbon scaffold. Hydrophilicity can facilitate the HER activity by lowering the adhesion force and facilitating the detachment of gas bubbles from the catalyst surface [35,43].

Figure 3b shows the Tafel plots of the prepared catalysts, fitted to Tafel equation (η = b log|J| + a, where b is the Tafel slope and J is the current density). The Tafel slope of Pt/C is 36 mV dec^−1^, which is consistent with the previously reported value [20]. Meanwhile, the Tafel slope of the Mo_2_C/N,S-C is 56 mV dec^−1^, which is smaller than that of the Mo_2_C/S-C (75 mV dec^−1^) and the c-Mo_2_C (110 mV dec^−1^), thereby suggesting the occurrence of the Volmer–Heyrovsky mechanism along with the faster HER kinetics in the Mo_2_C/N,S-C.

The Nyquist plots obtained from the electrochemical impedance spectroscopy (EIS) of the catalysts are presented in Figure 3c, where the charge transfer resistance (R_ct_) at the electrode and electrolyte interface is represented by a semicircle and is inversely proportional to the electrocatalytic activity [20,44]. The R_ct_ value of Mo_2_C/N,S-C (12.4 Ω), which is smaller than that of Mo_2_C/S-C (35.1 Ω) and c-Mo_2_C (200 Ω), thereby implies rapid electron transfer and improved HER activity.

The cyclic voltammogram (CV) curves of the catalysts in the non-faradaic region are provided in Figure S5a–c, and the corresponding double-layer capacitance (C_dl_) values are shown in Figure S5d. The Mo_2_C/N,S-C catalysts exhibited a higher C_dl_ value of 18.02 mF cm^−2^ than Mo_2_C/S-C (5.47 mF cm^−2^) and Mo_2_C (1.34 mF cm^−2^). In general, C_dl_ is proportional to the contact area between catalyst and electrolyte. Accordingly, the contact area of Mo_2_C/N,S-C is larger than other catalysts, which additionally contributes to the higher HER activity [25].

The polarization curves of the Mo_2_C/N,S-C obtained before and after 3000 cycles between 0.2 and −0.2 V (vs. RHE) are presented in Figure 3d. This reveals the good stability of the catalyst in alkaline media, with little change in the polarization curve, and a marginal increase in the η_10_ value from 105 to 117 mV, after 3000 cycles. Since durability is a significant factor in determining the HER performance, the highly active and durable Mo_2_C/N,S-C has clear potential as a HER electrocatalyst.

## 4. Conclusions

In summary, a simple and environmentally friendly method to produce Mo_2_C nanoparticles dispersed onto N,S co-doped carbon scaffold was designed by using the lignosulfonate, an industrial waste material. The as-prepared Mo_2_C/N,S-C catalyst exhibited a high HER performance with a small η_10_ value of 105 mV and a good stability for 3000 cycles. The improved HER performance resulted from a synergy between the highly active Mo_2_C nanoparticles and N,S co-doped carbon scaffold, thus providing high electrical conductivity and large surface area. Compared to solely doped carbon scaffolds, N,S-C scaffold can further enhance the HER performance of Mo_2_C by adjusting the catalytic activity of carbon atoms adjacent to heteroatoms. In addition, N,S co-doping to carbon modulated the hydrophilicity of the catalyst surface, thereby further enhancing the HER activity. Thus, considering this simple and environmentally friendly method, the proposed Mo_2_C/N,S-C could be a promising HER catalyst with a high activity and stability.

## Data Availability

All data is contained within the article.

## Supplementary Materials

The following supporting information can be downloaded at: https://www.mdpi.com/article/10.3390/nano12173047/s1, Figure S1: (a) TEM and (b) HR-TEM images for Mo_2_C/S-C; Figure S2: XPS survey scan of Mo_2_C/N,S-C; Figure S3: XPS spectra of Mo_2_C/S-C. (a) Survey, (b) Mo 3d (c) N 1s, and (d) S 2p; Figure S4: (a) N_2_-sorption isotherm and (b) pore size distribution of Mo_2_C/N,S-C; Figure S5: CV graphs of (a) Mo_2_C/N,S-C, (b) Mo_2_C/S-C and (c) Mo_2_C measured at scan rates from 20 to 180 mV s^−1^ between potential range of 0.1 and 0.3 V (vs. RHE) in 1 M KOH. (d) measured capacitive currents at 0.2 V (vs. RHE) as a function of scan rate; Table S1: Element contents of lignosulfonate; Table S2: The amounts of each element for Mo_2_C/N,S-C and Mo_2_C/S-C; Table S3: Comparison of HER performance in alkaline media with various reported biomass-derived molybdenum carbide-based catalysts. References [45,46,47,48,49,50,51,52,53].
