# Supplementary material for: Molybdenum Carbide Anchored on N,S Co-Doped Carbon Composite Derived from Lignosulfonate as a High Performance Electrocatalyst for Hydrogen Evolution Reaction
Source: Nanomaterials (Basel). 2022 Sep 2;12(17):3047. doi: 10.3390/nano12173047 (PMC9458135; doi:10.3390/nano12173047)
Supplement: Supplementary file 1 [file nanomaterials-12-03047-s001.zip › nanomaterials-1883357-supplementary.pdf]

nanomaterials

Supplementary Materials

Molybdenum Carbide Anchored on N, S Co-Doped Carbon Composite Derived from Lignosulfonate as a High Performance Electrocatalyst for Hydrogen Evolution Reaction

Na Yeong Oh [1], So Young Park [1], Ji Young Hwang [1], Hyung Mo Jeong [2], Yong Sik Kim [3,*] and Duck Hyun Youn [1,*]

[1] Department of Chemical Engineering, Department of Integrative Engineering for Hydrogen Safety, Kangwon National University, Chuncheon 24341, Korea; 5nayeong@kangwon.ac.kr (N.Y.O.); parksy@kangwon.ac.kr (S.Y.P.); jyh_@kangwon.ac.kr (J.Y.H.)
[2] School of Mechanical Engineering, Department of Smart Fab. Technology, Sungkyunkwan University, Suwon 16419, Korea; hmjeong@skku.edu
[3] Department of Paper Science & Engineering, Kangwon National University, Chuncheon 24341, Korea
* Correspondence: yongsikk@kangwon.ac.kr (Y.S.K.); youndh@kangwon.ac.kr (D.H.Y.)

Citation: Oh, N.Y.; Park, S.Y.; Hwang, J.Y.; Jeong, H.M.; Kim, Y.S.; Youn, D.H. Molybdenum Carbide Anchored on N, S Co-Doped Carbon Composite Derived from Lignosulfonate as a High Performance Electrocatalyst for Hydrogen Evolution Reaction. *Nanomaterials* **2022**, *12*, 3047. https://doi.org/10.3390/nano12173047

Academic Editor: Antonino Gulino

Received: 09 August 2022
Accepted: 28 August 2022
Published: 2 September 2022

Publisher's Note: MDPI stays neutral with regard to jurisdictional claims in published maps and institutional affiliations.

Copyright: © 2022 by the authors. Licensee MDPI, Basel, Switzerland. This article is an open access article distributed under the terms and conditions of the Creative Commons Attribution (CC BY) license (https://creativecommons.org/licenses/by/4.0/).

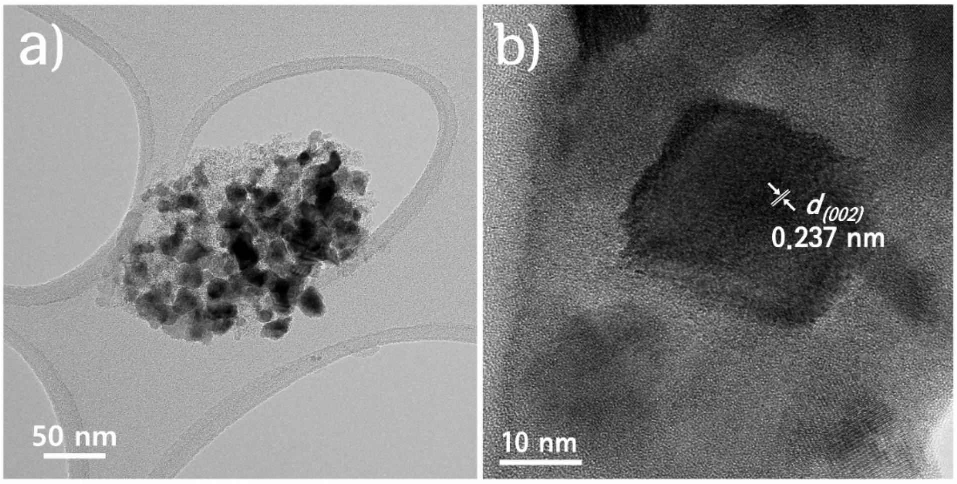

Figure S1. (**a**) TEM and (**b**) HR-TEM images for Mo_2C/S-C.

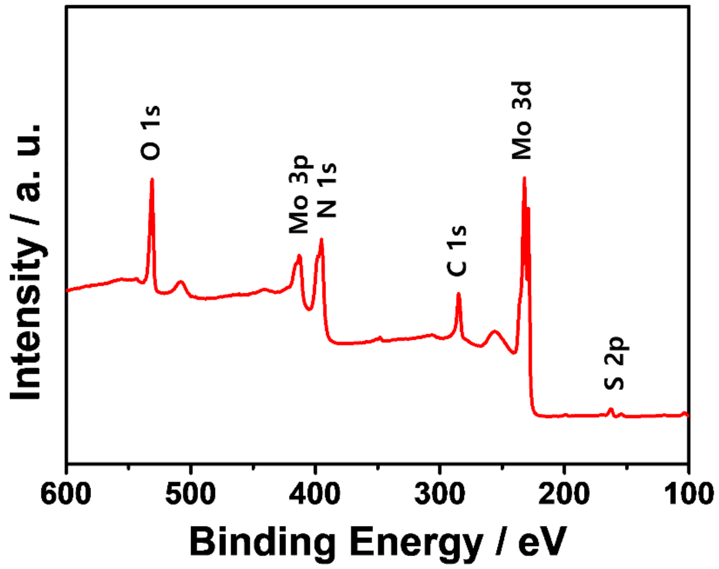

Figure S2. XPS survey scan of Mo_2C/N,S-C.

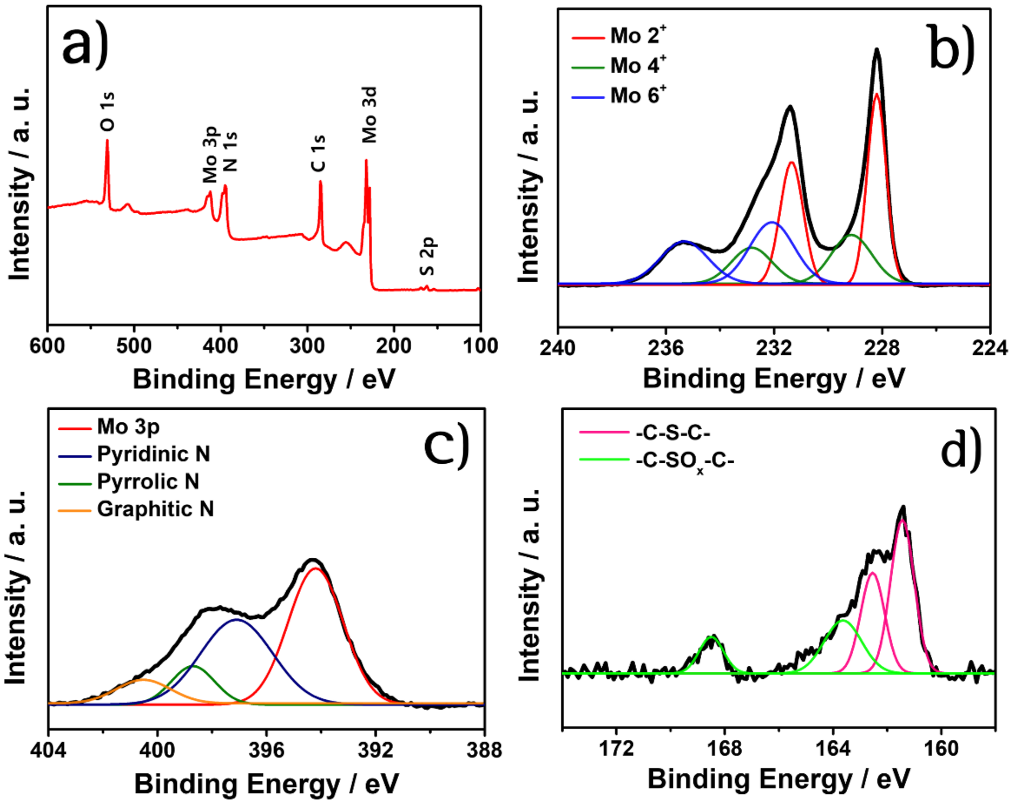

Figure S3. XPS spectra of Mo_2C/S-C. (**a**) Survey, (**b**) Mo 3d (**c**) N 1s, and (**b**) S 2p.

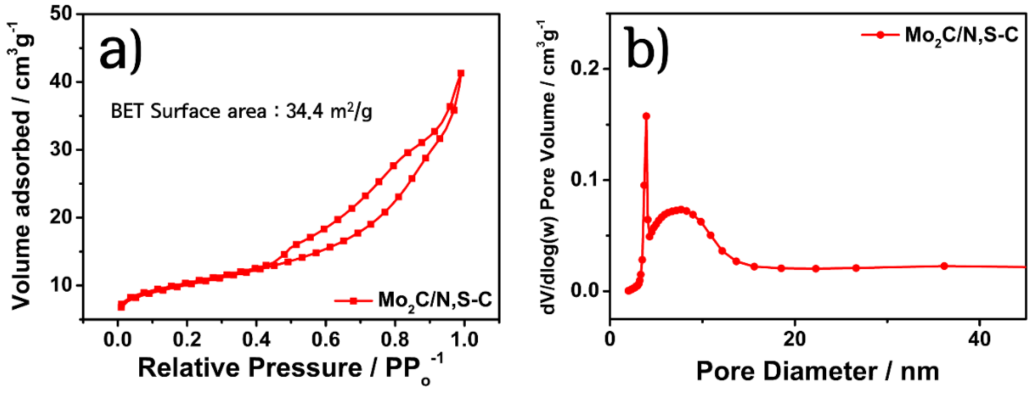

Figure S4. (**a**) N_2-sorption isotherm and (**b**) pore size distribution of Mo_2C/N,S-C.

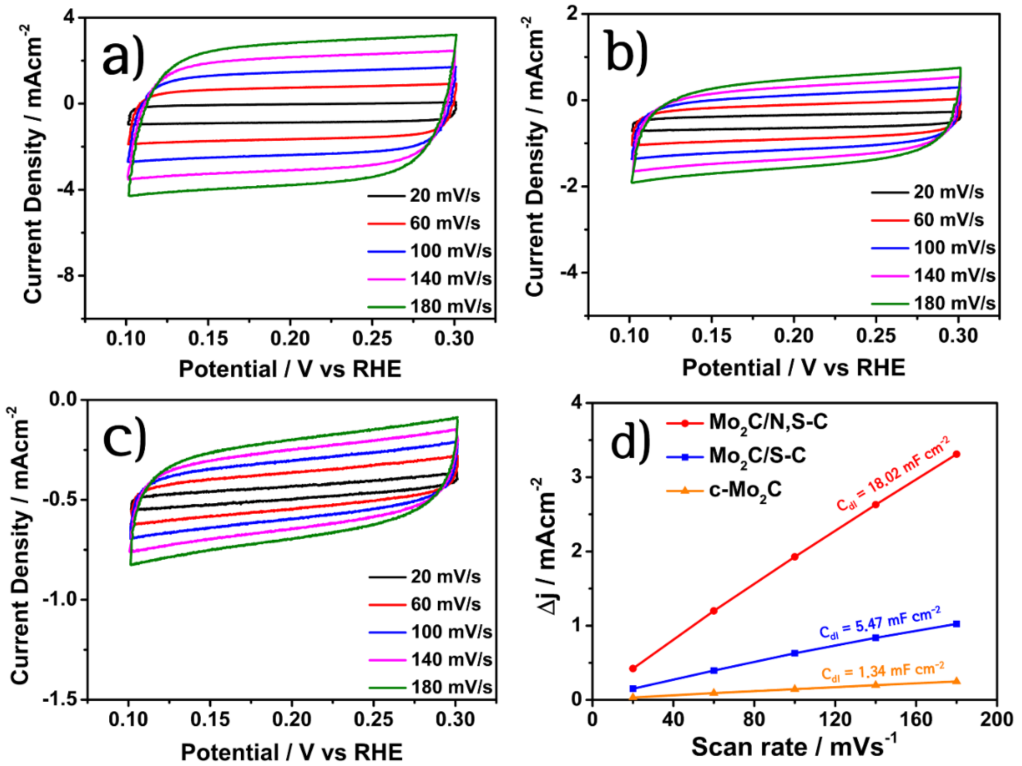

Figure S5. CV graphs of (**a**) Mo_2C/N,S-C, (**b**) Mo_2C/S-C and (**c**) Mo_2C measured at scan rates from 20 to 180 mV s^{-1} between potential range of 0.1 and 0.3 V (vs. RHE) in 1 M KOH. (**d**) measured capacitive currents at 0.2 V (vs. RHE) as a function of scan rate.

Table S1. Element contents of lignosulfonate.

**Sample**	**C (%)**		**N (%)**		**S (%)**	
	Average	Standard deviation	Average	Standard deviation	Average	Standard deviation
Lignosulfonate	45.9	0.3	2.1	0.0	3.0	0.0

Table S2. Comparison of HER performance in alkaline media with various reported biomass-derived molybdenum carbide-based catalysts.

**Biomass source**	**Catalyst**	η_{10} **(mV)**	**Tafel slope (mV/dec)**	**Ref.**
Lignosulfonate	Mo_2C/N,S-C	105	56	This work
Pomelo peels	Mo_2C/NKAB	144	53	[45]
Agaric	Mo_2C/N-CANs	100	75.9	[46]
Chitosan	Mo_2C QDs/NGCLs	111	57.8	[47]
Starch	Mo_xC/N-PC	100	94.5	[48]
Rice paper	Mo_2C@3DNMC	78	64	[49]
Sunflower seeds	Mo_2C@SNC	60	71	[50]
Bacterial cellulose	Mo_2C@N-CNF	168	47.1	[51]
leather	Mo_2C-0.4@CNCC	113	82.5	[52]
Lotus roots	MoO_2/Mo_2C/C	138	56.7	[53]

References

45. Guo, T.; Zhang, X.; Liu, T.; Wu, Z.; Wang, D., N, K Co-activated biochar-derived molybdenum carbide as efficient electrocatalysts for hydrogen evolution. *Appl. Surf. Sci.* **2020,** *509*, 144879.
46. Kang, Q.; Li, M.; Wang, Z.; Lu, Q.; Gao, F., Agaric-derived N-doped carbon nanorod arrays@nanosheet networks coupled with molybdenum carbide nanoparticles as highly efficient pH-universal hydrogen evolution electrocatalysts. *Nanoscale* **2020,** *12*, 5159-5169.
47. Pu, Z.; Wang, M.; Kou, Z.; Amiinu, I. S.; Mu, S., Mo_2C quantum dot embedded chitosan-derived nitrogen-doped carbon for efficient hydrogen evolution in a broad pH range. *Chem. Commun.* **2016,** *52*, 12753-12756.
48. Han, W.; Chen, L.; Ma, B.; Wang, J.; Song, W.; Fan, X.; Li, Y.; Zhang, F.; Peng, W., Ultra-small Mo_2C nanodots encapsulated in nitrogen-doped porous carbon for pH-universal hydrogen evolution: insights into the synergistic enhancement of HER activity by nitrogen doping and structural defects. *J. Mater. Chem.* **2019,** *7*, 4734-4743.
49. An, K.; Xu, X., Mo_2C based electrocatalyst with nitrogen doped three-dimensional mesoporous carbon as matrix, synthesis and HER activity study. *Electrochim. Acta* **2019,** *293*, 348-355.
50. An, K.; Xu, X.; Liu, X., Mo_2C-based electrocatalyst with biomass-derived sulfur and nitrogen co-doped carbon as a matrix for hydrogen evolution and organic pollutant removal. *ACS Sustain. Chem. Eng.* **2018,** *6*, 1446-1455.
51. Wu, Z.-Y.; Hu, B.-C.; Wu, P.; Liang, H.-W.; Yu, Z.-L.; Lin, Y.; Zheng, Y.-R.; Li, Z.; Yu, S.-H., Mo_2C nanoparticles embedded within bacterial cellulose-derived 3D N-doped carbon nanofiber networks for efficient hydrogen evolution. *NPG Asia Mater.* **2016,** *8*, e288-e288.
52. Kang, Q.; Qin, Y.; Lu, Q.; Gao, F., Waste leather-derived (Cr, N)-co-doped carbon cloth coupling with Mo_2C nanoparticles as a self-supported electrode for highly active hydrogen evolution reaction performances. *J. Power Sources* **2020,** *476*, 228706.
53. Chen, X.; Sun, J.; Guo, T.; Zhao, R.; Liu, L.; Liu, B.; Wang, Y.; Li, J.; Du, J., Biomass-derived carbon nanosheets coupled with MoO_2/Mo_2C electrocatalyst for hydrogen evolution reaction. *Int. J. Hydrog. Energy* **2022.**
